# Activation of Endothelial Nitric Oxide (eNOS) Occurs through Different Membrane Domains in Endothelial Cells

**DOI:** 10.1371/journal.pone.0151556

**Published:** 2016-03-15

**Authors:** Jason Tran, Astrid Magenau, Macarena Rodriguez, Carles Rentero, Teresa Royo, Carlos Enrich, Shane R. Thomas, Thomas Grewal, Katharina Gaus

**Affiliations:** 1 EMBL Australia Node in Single Molecule Science, School of Medical Sciences, University of New South Wales, Sydney, Australia; 2 ARC Centre of Excellence in Advanced Molecular Imaging, University of New South Wales, Sydney, Australia; 3 Departament de Biologia Cel·lular, Immunologia i Neurociències, Facultat de Medicina, Universitat de Barcelona, 08036 Barcelona, Spain; 4 Centre de Recerca Biomèdica CELLEX, Institut d’Investigacions Biomèdiques August Pi i Sunyer (IDIBAPS), 08036 Barcelona, Spain; 5 School of Medical Sciences, The University of New South Wales, Sydney, Australia; 6 Faculty of Pharmacy A15, University of Sydney, Sydney, Australia; University of British Columbia, CANADA

## Abstract

Endothelial cells respond to a large range of stimuli including circulating lipoproteins, growth factors and changes in haemodynamic mechanical forces to regulate the activity of endothelial nitric oxide synthase (eNOS) and maintain blood pressure. While many signalling pathways have been mapped, the identities of membrane domains through which these signals are transmitted are less well characterized. Here, we manipulated bovine aortic endothelial cells (BAEC) with cholesterol and the oxysterol 7-ketocholesterol (7KC). Using a range of microscopy techniques including confocal, 2-photon, super-resolution and electron microscopy, we found that sterol enrichment had differential effects on eNOS and caveolin-1 (Cav1) colocalisation, membrane order of the plasma membrane, caveolae numbers and Cav1 clustering. We found a correlation between cholesterol-induced condensation of the plasma membrane and enhanced high density lipoprotein (HDL)-induced eNOS activity and phosphorylation suggesting that cholesterol domains, but not individual caveolae, mediate HDL stimulation of eNOS. Vascular endothelial growth factor (VEGF)-induced and shear stress-induced eNOS activity was relatively independent of membrane order and may be predominantly controlled by the number of caveolae on the cell surface. Taken together, our data suggest that signals that activate and phosphorylate eNOS are transmitted through distinct membrane domains in endothelial cells.

## Introduction

The plasma membrane is organised into distinct domains that have are thought to have a characteristic lipid composition and contain a subset of membrane proteins [1]. Such compartmentalization may be critical in the regulation of signalling pathways [2]. The most prominent lipid domains, lipid rafts, are defined as small, transient structures in the plasma membrane that are enriched in cholesterol and glycosphingolipids [1]. Originally identified as detergent resistant membranes (DRM) [3], glycosylphosphatidylinositol (GPI)-anchored proteins, acylated proteins and selected transmembrane proteins [1, 2] are proposed to be associated with lipid rafts due to the preferential partitioning into highly ordered regions of reconstituted [4–8] and cellular membranes [9, 10]. In reconstituted membranes, cholesterol and sphingolipids are able to promote phase separation between liquid-ordered (l_o_) and liquid-disordered (l_d_) phases [11]. Hence, the biophysical hallmark of lipid raft is a high membrane order, which can be quantified with the fluorescent lipid dye, 6-lauroyl-propiony-2-dimethylamino-naphthalene (Laurdan) and two-photon microscopy [12, 13].

Caveolae are specialised plasma membrane domains containing the integral membrane protein caveolin-1 [1, 14]. They are classified as relatively small (50–100 nm), flask-shaped invaginations of the plasma membrane [15]. Isolation of caveolin-rich membranes by detergent resistant methods led to the identification of a number of proteins associated with caveolae such as the class B scavenger receptors CD36 and SR-BI for modified low-density lipoprotein (LDL) and high-density lipoprotein (HDL), respectively, as well as GPI-linked proteins and multiple cytoplasmic signalling molecules [16, 17].

One of the key functions of endothelial cells is the production of nitric oxide (NO), and the enzyme responsible for NO production is endothelial nitric oxide synthase (eNOS). In endothelial cells, eNOS generates NO in the reaction converting L-arginine to L-citrulline [18]. The endothelial isoforms of NOS bind calmodulin (CaM) in a calcium (Ca^2+^)-dependent manner and can be activated by diverse extracellular stimuli including vascular endothelial growth factor (VEGF), HDL, shear stress and pharmacological agents that increase intracellular Ca^2+^ [19, 20].

eNOS localises to the plasma membrane [19], the Golgi complex [21], the cytosol, mitochondria and the nucleus [22]. At the plasma membrane, eNOS association with caveolae and non-caveolar domains within the plasma membrane was shown to be dependent on its palmitoylation, myristoylation and phosphorylation [23, 24]. eNOS also interacts with Cav1 independently of the acylation state of the enzyme [25] and Cav1 negatively regulates eNOS in caveolae [26]. In particular the latter studies, using rat thyroid and prostate cancer cell lines, provided the first example of spatial regulation of signalling in caveolae that was distinct from non-caveolar raft domains [26]. Residues 82–101 in the scaffolding domain of Cav1 have been proposed to bind eNOS inhibiting the interaction of the enzyme with Ca^2+^-CaM [27, 28] although the details of the interaction have been questioned [29]. *In vivo* studies showed that over-expression of Cav1 in the endothelial layer inhibited VEGF-mediated activation of eNOS [30]. Conversely, Cav1-deficient mice had increased eNOS activity and systemic levels of NO [31]. These studies suggest that subcellular localization of eNOS regulates its activity and is at least partially governed by Cav1 expression levels.

Despite all of the aforementioned knowledge on microdomains contributing to eNOS activation [19–31], a comparison of how cholesterol enrichment in endothelial cells impacts on the ability of membrane domains to transmit eNOS activating signals after stimulation with VEGF, HDL or shear stress, is still lacking. In endothelial cells, VEGF binds to VEGF receptors (VEGFR2, also known as KDR/Flk-1) that localise to caveolae and associate with Cav1 [32] and eNOS [33]. Cav1 negatively regulates VEGFR2 in non-stimulated conditions. Stimulation with VEGF results in the rapid dissociation of VEGFR2 and Cav1 from caveolae [32]. Further, there is evidence that activated VEGFR2 utilizes signalling protein complexes at focal adhesions, enriched with highly ordered membrane domains [34] to initiate biological responses [35, 36].

HDL-mediated stimulation of eNOS activation occurs through HDL binding to SR-BI, which associates with DRM and Cav1 [17, 37]. While the majority of current literature favours a localization of SR-BI in lipid rafts and caveolae [38] the precise cellular localization of SR-BI at the cell surface is not fully understood [39]. Binding of HDL to SR-BI can facilitate the exchange of cholesterol and phospholipids between the plasma membrane and the lipoprotein particle, which could modulate membrane domains and indirectly the localization of SR-BI. Most relevant to HDL-induced activation of eNOS, the ability of SR-BI to bind cholesterol and capacity to facilitate cholesterol flux is a pre-requisite to initiate signal transduction cascades needed for eNOS activation [38, 40, 41].

Shear stress forces are imposed directly on the endothelium by the circulating blood and NO derived from eNOS is one of the major regulators of vessel reorganisation in response to shear stress. Haemodynamic forces release the inhibition of eNOS by Cav1 in caveolae [42] and increase its activity by allowing access to Ca^2+^-CaM [43]. *In situ* studies showed that intact caveolae at the luminal surface of endothelial cells were necessary for flow-induced activation of signalling pathways [43–45] involved in eNOS phosphorylation at Ser^1179^ [46, 47]. Conversely, chronic shear stress induces translocation of Cav1 from the Golgi to the plasma membrane and increases luminal caveolae formation suggesting that shear stress plays a role in caveolin-1/caveolae trafficking [47, 48].

Since cholesterol is an integral component of lipid raft domains and directly binds to Cav1, drastic cholesterol depletion using methyl-ß-cyclodextrin (mßCD) disrupts not only the plasma membrane organization but also results in the translocation of eNOS to intracellular pools, with subsequent effects on its activity. Rather than using drastic and unphysiological cholesterol depletion agents, and to better mimic fluctuations in cellular cholesterol levels without negatively affecting cell viability, here we compared the effects of enrichment of endothelial cells with cholesterol and the oxysterol 7-ketocholesterol (7KC) on the membrane organisation and activation of eNOS by VEGF, HDL and shear stress. 7KC is less effective than cholesterol in forming phospholipid-sterol complexes and protein-lipid complexes that support raft formation [49]. This is due to the location of the oxygen moieties and chiral nature of oxysterols, which limits their packing in phospholipid bilayers [50, 51]. We found that cholesterol and 7KC enrichment differentially affected plasma membrane order and caveolae, which allowed us to delineate the contributions of these domains to eNOS activation stimulated by VEGF, HDL and shear stress.

## Results and Discussion

BAEC cells were enriched with cholesterol (chol) or 7-ketocholesterol (7KC) complexed to methyl-ß-cyclodextrin (mßCD) for 30 min at 37°C. This resulted in a 1.6–2.2-fold increase in cellular sterol levels (Table 1). By gradually replacing cholesterol with 7KC in complexed with mßCD in solution, we achieved various cellular cholesterol to 7KC ratios, ranging from 0–30% 7KC of total cellular sterol concentration, while roughly maintaining the overall sterol levels. Although 7KC induces apoptosis at high concentrations [52], cell viability was >90% in all conditions (data not shown).

**Table 1:** Sterol composition of BAEC. BAEC were enriched with 40 μM Chol, 20 μM 7KC or a mixture of both 20 μM Chol and 20 μM 7KC (Ch:7KC). Sterols were extracted from total cell lysates and analysed by HPLC. Values are means ± S.D. of three independent experiments. n.d. = non detectable.

Condition	Cholesterol (nmol/mg)	7KC (nmol/mg)	7KC of total sterol (%)
Control	68.74 ± 18.1	n.d.	n.d.
Chol	118.03 ± 4.6	n.d.	n.d.
Ch:7KC	123.68 ± 24.2	30.5 ± 7.6	20%
7KC	73.1 ± 12.5	33.1 ± 0.6	30%

**Fig 1:**
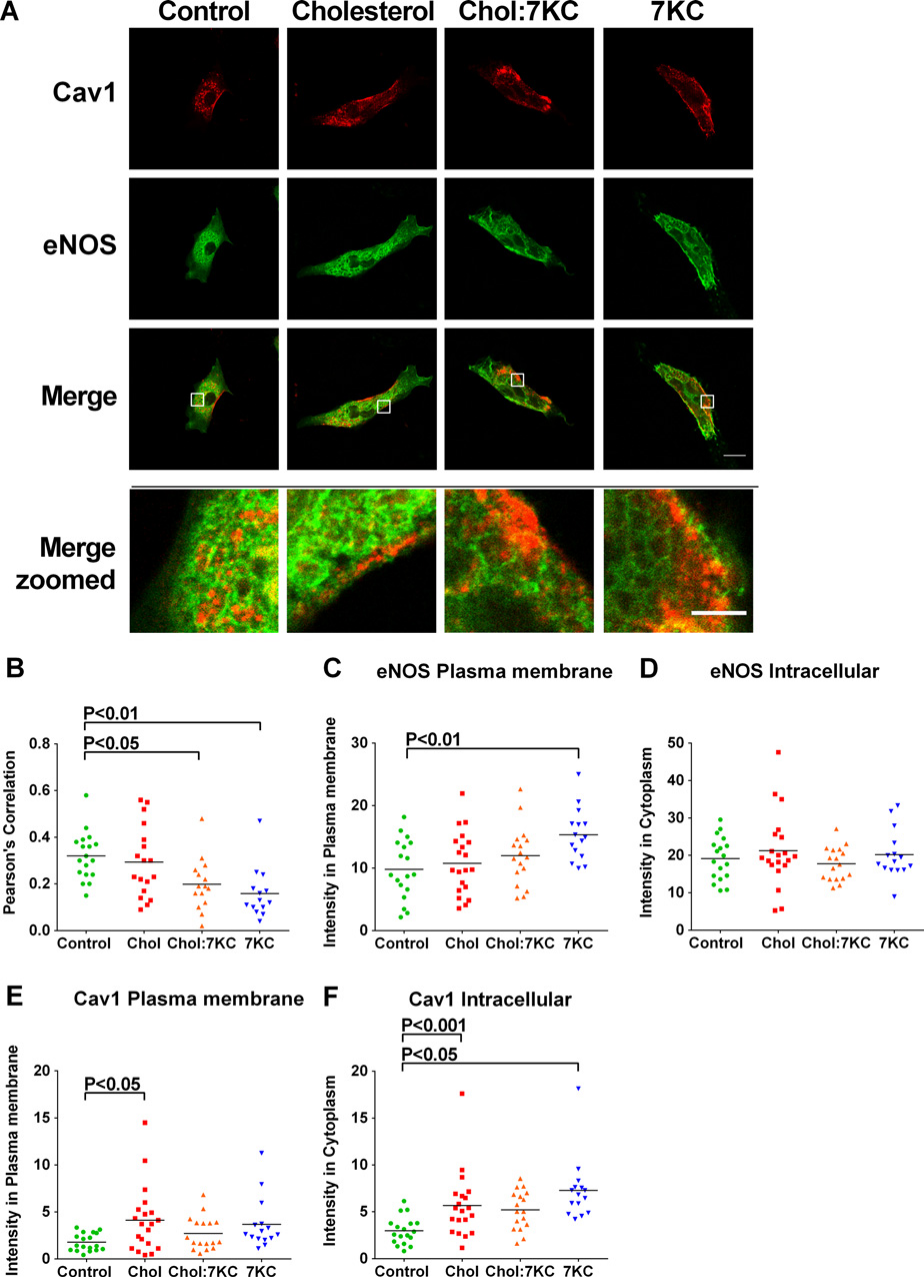
Immunofluorescence of sterol-manipulated BAEC. (A) BAEC plated on gelatin-coated coverslips were enriched with 40 μM Chol, 20 μM 7KC or a mixture of 20 μM Chol and 20 μM 7KC (Ch:7KC), fixed and immunostained with Cav1-Alexafluor 647 and eNOSIII-Alexafluor 488 and visualized by confocal fluorescence microscopy. Scale bar: 20 μM. (B) Pearson’s correlation of Cav1 and eNOS co-localization in confocal images. (C-F) Fluorescence intensity of eNOS (D-C) and Cav1 (E-F) in the plasma membrane (C, E) and in intracellular regions (D, F) in confocal images. The plasma membrane was defined as the region ~1.2 μm from the cell periphery and intracellular regions as adjacent to the plasma membrane regions. In C-F, each symbol represents one (merged) image and horizontal bars indicate means. Significant differences to control cells were tested with one-way ANOVA.

To examine Cav1 and eNOS protein distribution upon cholesterol or 7KC enrichment, we performed immunofluorescence imaging and quantified co-localization of Cav1 and eNOS by calculating the Pearson’s coefficient (Fig 1). While the overall protein expression was not affected by sterol treatment (Fig 1A), as confirmed by Western blotting (data not shown), the co-localization of Cav1 and eNOS was slightly reduced after 7KC enrichment (Fig 1B). We thus examined the subcellular distribution of eNOS and Cav1 in more detail by measuring the fluorescence intensity at the cell periphery *versus* intracellular intensity (Fig 1C–1F). We defined ‘plasma membrane’ as regions that were 5 pixels (= 1.2 μm) from the cell edge and ‘intracellular regions’ adjacent to the plasma membrane regions but further from the cell edge. 7KC enrichment but not enrichment in cholesterol resulted in an increased localisation of eNOS at the plasma membrane without alteration to intracellular staining intensity (Fig 1C and 1D). In contrast, cholesterol enrichment increased plasma membrane and intracellular staining of Cav1 while 7KC enrichment only elevated intracellular Cav1 (Fig 1E and 1F). However, the cholesterol-induced redistribution of Cav1 appeared to have no effect on Cav1-eNOS co-localization while the 7KC-induced increase of plasma membrane eNOS and intracellular Cav1 may have contributed to the reduction of Cav1-eNOS co-localization in these cells (Fig 1B).

**Fig 2:**
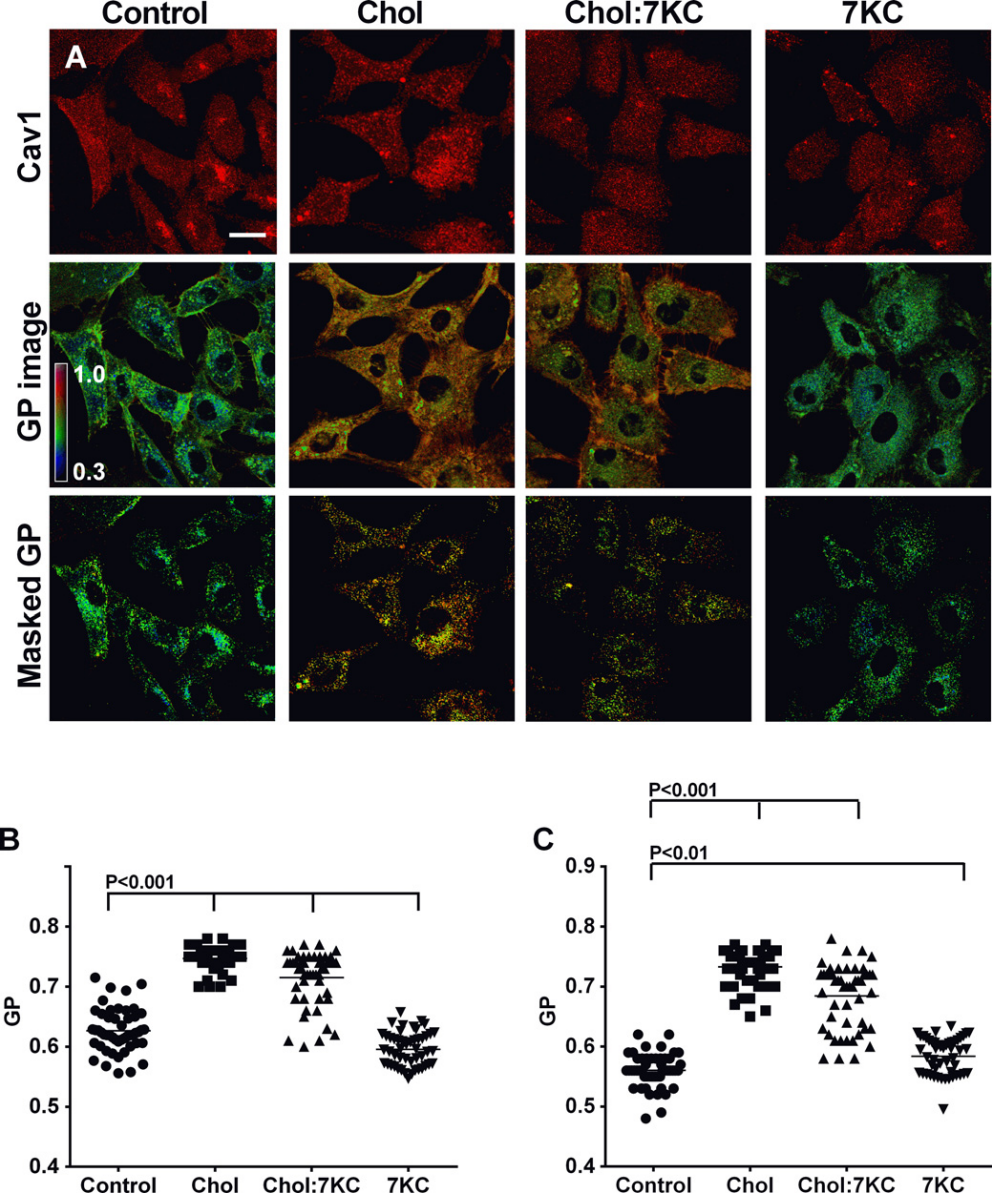
Effect of sterol enrichment on BAEC membrane order. BAEC were enriched with 40 μM Chol, 20 μM 7KC or a mixture of 20 μM Chol and 20 μM 7KC (Ch:7KC) and stained with 5 μM Laurdan followed by fixation and immunostaining for Cav1. (A) Confocal image of Cav1-Cy3 staining (top row), Laurdan GP image of the same focal depth (middle row) and masked GP images showing only the GP image of Cav1-positiv membrane areas (bottom row). GP images were pseudo-coloured according to the colour scale shown in the first GP image. Blue colour indicates GP values of 0.3 and red colour indicates GP values of 1.0. Scale bar = 20 μm. (B) Total plasma membrane GP values were determined from GP images recorded at high magnification. All sterol enrichment conditions were significantly different (P<0.001) to control cells. (C) Cav1-positive membrane GP values were determined using the masked GP image. Cholesterol and Chol:7KC enrichment increased membrane order significantly (P<0.001), while 7KC-treatment also increased membrane order compared to control (P<0.01). Data are from three independent experiments with a total of 60 cells. In B-C, each symbol is one image, typically with one cell; horizontal lines indicate means. Significances were tested with one-way ANOVA.

We next examined whether cholesterol and 7KC enrichment had an effect on the order of the plasma membrane using the fluorescent dye Laurdan and 2-photon microscopy. Laurdan is an environment-sensitive membrane probe whose emission spectrum reports the degree of lipid packing in the bilayer [53]. The membrane dye is not fluorescent in water and when incorporated into membrane shifts its emission spectra according to the polarity of the membrane. For example, in raft-like liquid-ordered membranes, lipids in the membrane are more closely packed together preventing the penetration of water molecules into the bilayer. This results in a blue-shifted Laurdan emission and a high Generalised Polarization (GP) value. Although other factors, such as Laurdan interacting with cellular transmembrane proteins cannot be excluded, Laurdan GP values are considered an excellent tool to determine membrane order. We thus recorded Laurdan intensity in two spectral channels and calculated the GP value for each pixel. GP values range from +1 (highly ordered membranes) to -1 (very fluid membranes) [34, 53–55]. To visualise membrane order, we generated pseudo-coloured GP images of control and sterol-manipulated BAEC with green indicating fluid membranes and red highly ordered membranes (Fig 2A, GP image). In line with previous data [54], cholesterol enrichment substantially increased the membrane order of BAEC, while 7KC enrichment reversed this effect. To identify caveolae membrane structures, Laurdan-labelled cells were immuno-stained for Cav1 and confocal images taken at the identical focal depth as the GP images (Fig 2A, Masked GP). The Cav1 confocal images were then used to mask the GP images so that only Cav1-positive pixel remained. Comparison to the GP images with the masked GP images shows that Cav1-positive pixels are found in predominantely highly ordered membranes.

To quantify membrane order of the plasma membrane from GP images and masked GP images, the plasma membrane was again defined as the cell perimeter ~1.2 μm (5 pixels) from the cell perimeter in high magnification images (data not shown). We then measured the GP values of total plasma membrane (Fig 2B) and of Cav1-positive plasma membrane regions (Fig 2C) and found little differences between these domains. This is not surprising given that both caveolae and lipid rafts are likely to be below the resolution limit of the microscope so that Cav1-positive plasma membrane domains may also contain non-raft domains and *vice versa*. However, we found a significant difference between the cholesterol and 7KC enriched conditions. Cholesterol enrichment significantly enhanced membrane order by increasing the abundance of raft-like domains. Given the resolution limit of the microscopy approach, we cannot discriminate whether an increase in raft abundance was caused by an increase in the number of raft-like domains, i.e. caveolae, or by enlarged ordered domain, increasing membrane order or a combination of these processes. 7KC reversed the cholesterol enrichment effect in a concentration-dependent manner so that 7KC-enriched cells had a lower plasma membrane order compared to control cells (Fig 2B). In contrast Cav1-positive membrane domains, maintained a higher membrane order even after enrichment with 7KC (Fig 2C), highlighting the ordering effect of Cav1 on its local lipid environment that may be masked in cholesterol-enriched membranes that are already highly condensed.

**Fig 3:**
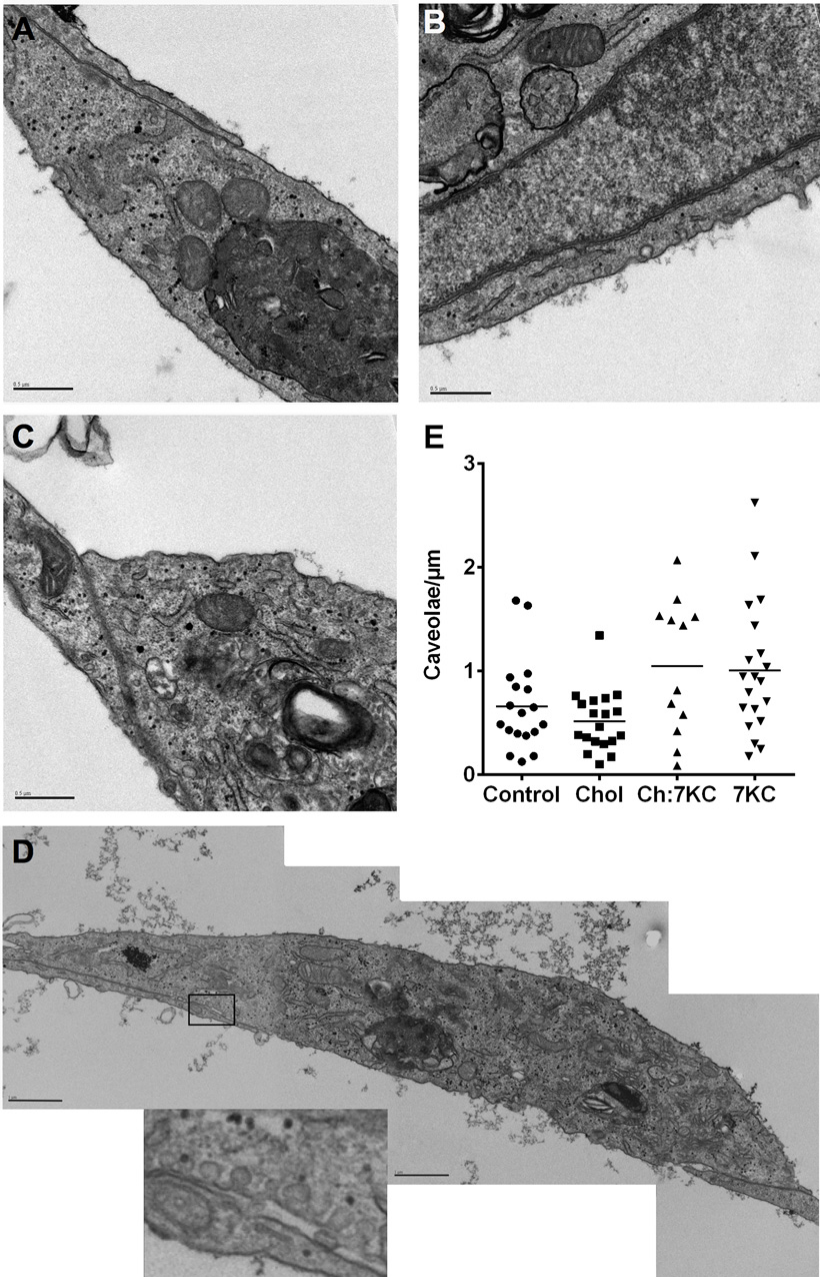
Caveolae quantification in control and sterol-enriched BAEC. BAEC were enriched with 40 μM Chol, 20 μM 7KC or a mixture of both 20 μM Chol and 20 μM 7KC (Ch:7KC). Cells were fixed and embedded in Spur resin, processed for TEM, and imaged. The images were connected using the plug-in Mosaic J in Image J. Representative TEM images of (A) control, (B) Chol, (C) Ch:7KC, and (D) 7KC-enriched cells. In (D), images were merged and a zoomed region is shown with caveolae visible in the membrane. Images are representative of >20 cells per condition. Scale bar = 0.5 μm (A-C) and 1 μm (D). (E) Caveolae was quantified along the plasma membrane to obtain the number of caveolae/μm of cell perimeter. Each symbol represents one cell and several images. Data were from 5 independent experiments. Horizontal lines indicate means. No significant difference was observed between control cells and sterol enriched cells (tested with one-way ANOVA).

Next, we examined the effect of sterol enrichment on the number of cell surface caveolae. Therefore, thin sections of fixed and embedded BAEC were imaged at 35,000–40,000-fold magnification by transmission electron microscopy (TEM, Fig 3). At this magnification, caveolae are easily identified by their characteristic morphology (see insert in Fig 3D). Representative TEM images of control (A), Chol (B), Ch:7KC (C), and 7KC-enriched (D) cells are shown. We stitched individual images together, counted the number of caveolae at the cell surface and normalised the number per μm of cell perimeter (Fig 3E). As evident from the quantification, there was considerable cell-to-cell variation with increased 7KC concentrations enlarging this viability (distribution of data points in Fig 3E). There was a trend towards higher caveolae density in 7KC-enriched cells but this was not statistical significant. Thus we concluded that sterol treatment had no effect on the number of caveolae at the cell surface despite the increase in Cav1 in the plasma membrane (Fig 1E) and the modulation of the membrane order by the sterols (Fig 2).

**Fig 4:**
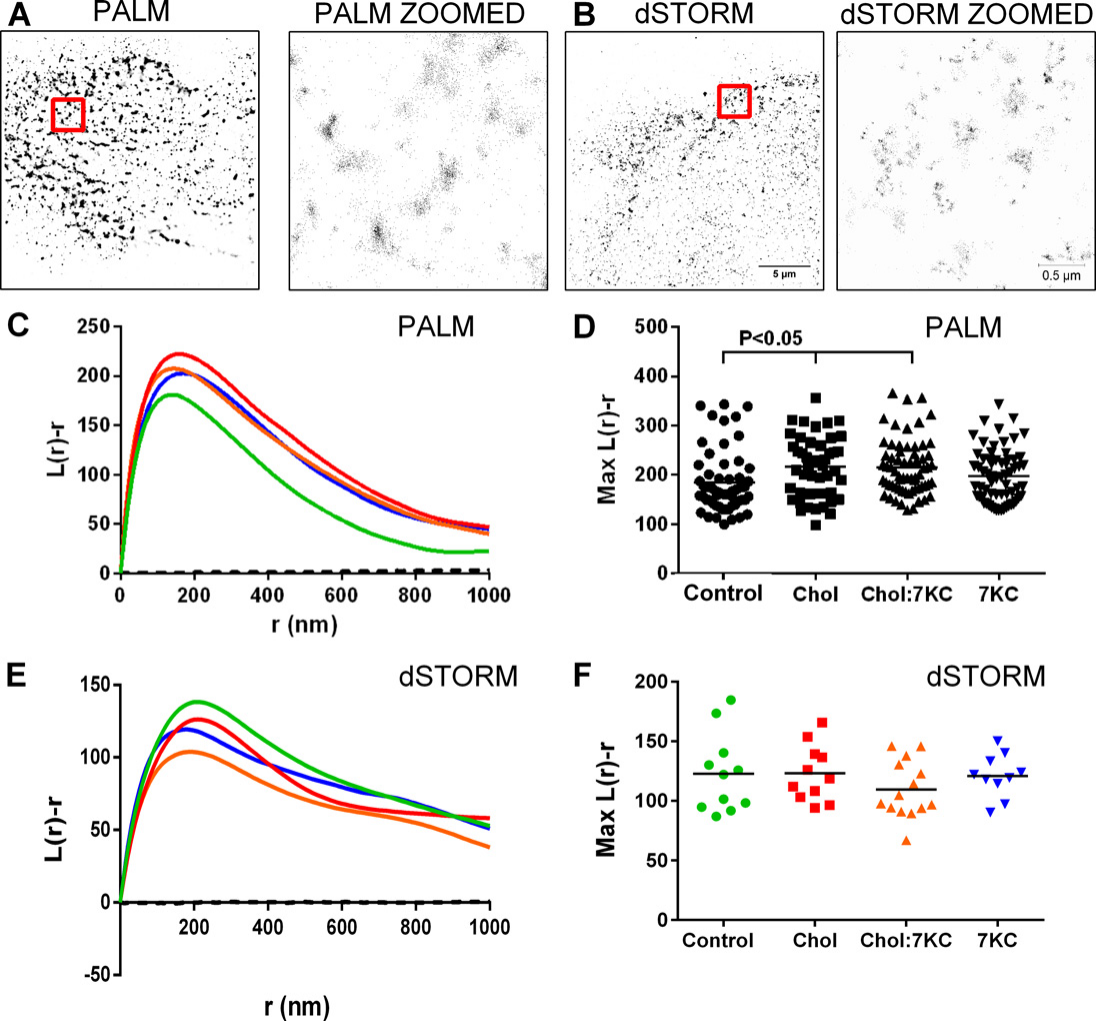
Quantification of Cav1 distributions by PALM and dSTORM. BAEC were transfected with Cav1-mEOS2 for PALM imaging (A, C-D) or immuno-stained with Cav1-Alexa Fluor 647 for dSTORM imaging (B, E-F). BAEC were left untreated (control) or treated with sterols as indicated, fixed and imaged by PALM and dSTORM. (A-B) PALM (A) and dSTORM (B) TIRF images of Cav1 in the membrane of BAEC (scale bar = 5 μm) with the white square indicating the zoomed region (scale bar = 0.5 μm). Each black dot represents one detected Cav1 molecule. (C-F) Ripley’s K-function plots (C, E) and quantification (D,F) of Cav1 distribution of PALM (C-D) and dSTORM (E-F) images. The L(r)-r reports the degree of non-randomness in point patterns on the radial length scale, r, relative to a random distribution (indicated by the dotted lines). In C and E, Ripley’s K-function plots of control (green), cholesterol- (red), Chol:7KC- (orange) and 7KC-treated cells (blue) are shown. In D and F, the maximum L(r)-r values reflect the maximum degree of clustering. Each symbol is one image region; horizontal bars reflect means. Significances were calculated with one-way ANOVA (no significant differences were found in F).

To evaluate the distribution of Cav1 in the membrane on the molecular level, we made use of photoactivated localisation microscopy (PALM) in combination with quantitative cluster analysis [56, 57]. Cells were transfected with Cav1 tagged to the photoconvertible protein mEOS2 (Cav1-mEOS2), which converts from green to red upon irradiation with a UV laser. Transfected cells were treated with sterols as described above and the red-converted form was imaged under total internal reflection (TIRF) restricting the excitation zone to ~150 nm beyond the glass-cell membrane interface. Single molecules were detected by fitting a point-spread-function (PSF) to each fluorescent event and calculating the centre of each PSF. This allowed the direct localisation of Cav1 molecules with ~20 nm precision (Fig 4A) and the quantification of Cav1 clustering with Ripley’s K-function analysis (Fig 4C and 4D). An example for a single molecule image of Cav1-mEOS2 is shown in Fig 4A. The Ripley K function, L(r)-r measures the molecular distribution relative to a spatially random distribution [58, 59]. The length scale, r, at which L(r)-r peaks indicates the dominant scale of Cav1 clustering, which was similar for all cellular conditions (Fig 4C). To quantify whether sterol enrichments altered the molecular distribution of Cav1, we extracted the maximum value of L(r)-r for individual images. With Cav1-mEOS2, we found an increase in Cav1 clustering in cholesterol-enriched cells, a trend that was again reversed by 7KC enrichment (Fig 4D).

To test whether overexpression of Cav1 in conjunction with cholesterol enrichment may have caused the enhanced clustering of Cav1, we also used direct stochastic optical reconstruction microscopy (dSTORM) where endogenous Cav1 is probed for by immuno-staining (Fig 4B). We achieved a similar localisation precision as with PALM. The Ripley K function curves peaked at a similar length scale for endogenous Cav1 as they did for Cav1-mEOS2 (Fig 4E). However, it was noticeable that the maximum degree of clustering (max L(r)-r values) was lower with dSTORM compared to PALM (Fig 4F). Indeed, we found no differences in the maximum L(r)-r values in sterol-treated cells. This is an interesting observation, possibly indicating differential impact of cholesterol enrichment on the distribution of endogenous Cav1 and ectopically expressed Cav1 fused to mEOS2. Indeed, recent studies showed that overexpressed Cav1 fusion proteins enhanced the aggregation and degradation of wild-type Cav1 [60], suggesting that overexpressed Cav1 fusion proteins and endogenous Cav1 may localize and behave differently. Although beyond the scope of this study, future research may reveal the impact of protein tags on and overexpression of Cav1 on the cellular distribution of endogenous Cav1. Taken together, the data suggest that overexpression of Cav1 enhances Cav1 clustering in a cholesterol-assisted manner. Similar to Cav1 fluorescence intensity in the plasma membrane (Fig 1F) and caveolae densities (Fig 3F), it appears that sterol-enrichment did not lead to a redistribution of Cav1 at the cell surface.

**Fig 5:**
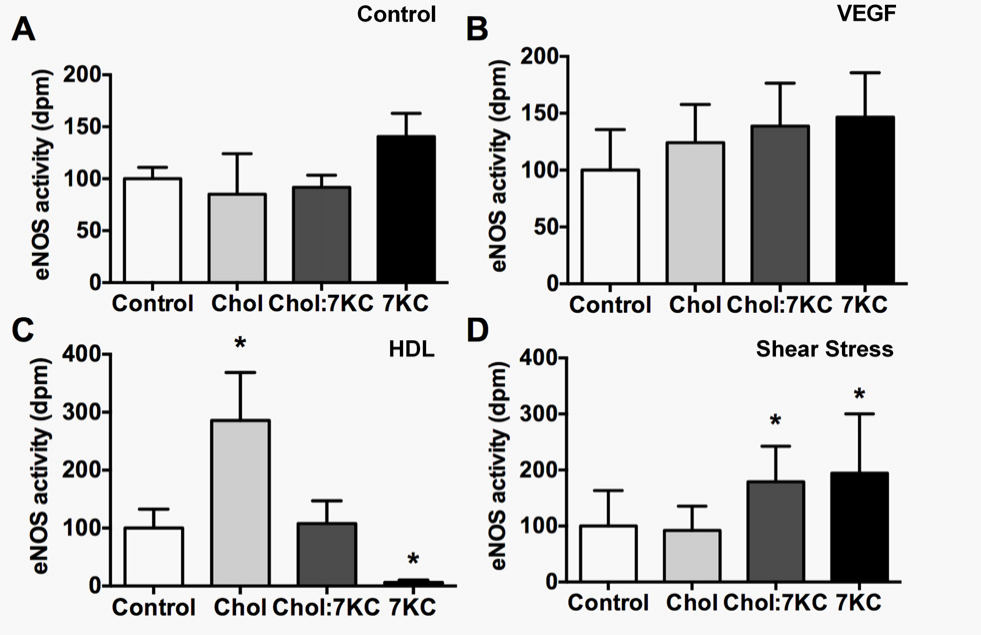
eNOS activity of sterol-manipulated BAEC in response to various stimuli. BAEC were enriched with 40 μM Chol, 20 μM 7KC or a mixture of both (20 μM Chol:20 μM 7KC). Cells were left untreated (control, A) or stimulated with 25 ng/ml VEGF for 5 min (B), 50 μg/ml HDL for 20 min (C) or with 10 dynes/cm^2^ shear stress for 5 min (D). Data is reported as the extent of conversion of [^3^H]-L-arginine to [^3^H]-L-citrulline that is sensitive to pre-treatment of endothelial cells for 30 min with 1 mM L-NAME and is expressed as dpm/10^6^ cells. Data were normalised to control and are expressed as means ± SEM from at least seven independent experiments performed in triplicate. Significance compared to control was calculated by one-way ANOVA and one asterisk indicates P<0.05.

The differential effect of sterol enrichment on membrane domains in endothelial cells gave us the opportunity to investigate the sensitivity of eNOS activation upon stimulation with VEGF, HDL and shear stress. As we were interested in the early signal transduction pathways that are possibly mediated by distinct membrane domains, we incubated BAEC with 25 μg/mL VEGF for 5 min, or 50 μg/mL HDL for 20 min or exposed BAEC to 10 dynes/cm^2^ shear stress for 5 min and compared eNOS activity to stimulated control cells (Fig 5). The expression of VEGFR2, SR-B1 as well as Src kinase and phosphorylation of extracellular signal-regulated kinase 1/2 (ERK1/2) were not affected by the sterol treatments as determined by Western blotting (data not shown). eNOS activity was quantified by measuring the conversion of [^3^H]-L-arginine to [^3^H]-L-citrulline in the presence or absence of the specific eNOS inhibitor, L-NAME. All three stimuli–VEGF, HDL and exposure to shear stress—induced eNOS activity in endothelial cells 5-10-fold over non-stimulated cells while sterol modifications without additional stimuli did not alter basal eNOS activity (data not shown).

**Fig 6:**
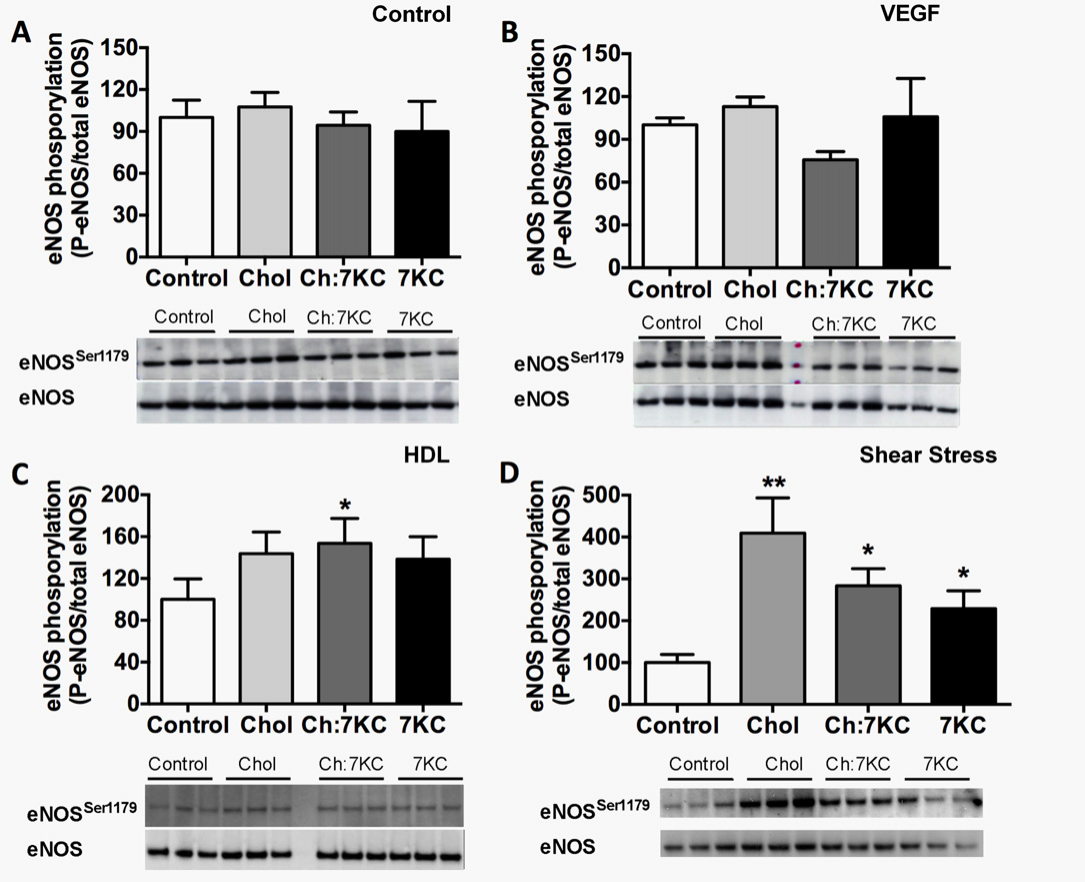
eNOS phosphorylation of sterol-manipulated BAEC in response to various stimuli. BAEC were enriched were treated as above and then either not stimulated (A) or stimulated with VEGF (25 ng/ml) for 5 min (B), 50 μg/ml HDL for 20 min (C) or stimulated with 10 dynes/cm^2^ shear stress for 5 min (D). Cells were lysed and equal protein amounts analysed by immunoblotting with antibodies against phospho-eNOS^Ser1179^ and total eNOS. Phosphorylation was measured with densitometry of phosphorylated protein divided by the total protein and normalised to control conditions. Data represent means ± SEM from six independent experiments. Significance compared to control was calculated by one-way ANOVA and is indicated by one asterisk reflecting P<0.05 or two asterisks indicating P<0.01.

eNOS activity was similar in control and sterol-manipulated BAEC with and without VEGF activation (Fig 5A and 5B). There was a trend towards increased eNOS activity upon 7KC enrichment in control and VEGF incubated cells, possibly indicating more eNOS at the plasma membrane (Fig 1D) and loss of the inhibitory eNOS-Cav1 interaction (Fig 1B). However, these findings were statistically not significant and mirrored when cell lysates of VEGF-stimulated and non-stimulated cells were probed for eNOS phosphorylation at Serine 1179 (Fig 6A and 6B), showing that sterol enrichment had no major effect on basal and VEGF-induced eNOS activity and phosphorylation. In contrast, when BAEC were stimulated with HDL (Fig 5C), eNOS activity was significantly enhanced in cholesterol-enriched cells while increasing levels of 7KC reduced the cholesterol-enhanced eNOS activity. These findings could indicate that cholesterol, but not 7KC, enrichment, improves HDL binding to SR-BI, enabling more rapid and efficient cholesterol flux, which is considered essential for eNOS activation. This change in eNOS activity was associated with a 1.5-fold increase in eNOS phosphorylation in cholesterol-enriched cells (Fig 6C). Interestingly, elevated eNOS phosphorylation was also observed upon increasing 7KC levels (Fig 6C). The similarity between data sets obtained for membrane order (Fig 2) and HDL-induced eNOS activity (Fig 5C) suggests that cholesterol and 7KC-induced changes in membrane order, rather than eNOS-Cav1 interaction or the number of caveolae, have the potential to affect eNOS activity. Under shear stress conditions, eNOS activity was significantly elevated in Chol:7KC- and 7KC-enriched cells (Fig 5D). In contrast, eNOS phosphorylation (Fig 6D) again was distinctly different from the pattern of eNOS activity (Fig 5D), peaking in cholesterol-enriched cells and being reduced by 7KC addition. Taken together, we found two stimulatory eNOS conditions, HDL and shear stress, to be highly sensitive to cholesterol or 7KC enrichment, leading to eNOS phosphorylation patterns at Ser1179 that did not reflect eNOS activity.

## Conclusion

We demonstrated that in endothelial cells, exogenously added cholesterol and 7KC differentially affect the subcellular distribution of Cav1 and eNOS, as well as the membrane order, but not the overall number of caveolae at the cell surface. These changes are associated with a differential response to VEGF-, HDL-, or shear stress-induced eNOS phosphorylation and activity, supporting a model that depending on the stimuli, different microdomains at the plasma membrane contribute to regulate eNOS activation.

Although 7KC, but not cholesterol enrichment, increased eNOS localization at the plasma membrane and reduced Cav1-eNOS co-localization, this did not appear to contribute to significant changes in eNOS phosphorylation and activity in basal and VEGF-stimulated conditions. Hence, we hypothesize that release of eNOS from Cav1 does not automatically activate eNOS although eNOS-Cav1 binding inhibits eNOS enzymatic activity. Given the large amounts of VEGFR2 in caveolae, it is tempting to speculate that VEGF-induced eNOS activation is linked to caveolae numbers rather than increased formation of highly ordered domains.

However, the differential response observed for HDL- and shear stress-induced eNOS activity and phosphorylation implicates that changes in membrane order may have substantial consequences depending on the stimuli analyzed. Here, the cholesterol-mediated effect on membrane organisation correlating with enhanced eNOS activity and phosphorylation in HDL-incubated endothelial cells are particularly striking. This suggests that membrane order generally or cholesterol-enhanced domains in the plasma membrane, rather than eNOS-Cav1 interaction or caveolae *per se*, influence HDL-induced eNOS activation. We speculate that an improved ability of SR-BI to bind cholesterol, possibly enabling a more targeted cholesterol flux between HDL and cholesterol-enriched plasma membrane domains, could promote signal transduction to more effectively enable eNOS activation. In support of this model, 7KC enrichment would interfere with the proposed cholesterol sensing role of SR-BI in this context, completely abrogating eNOS activity, yet still allowing eNOS phosphorylation.

The findings above indicated that some signalling cascades that trigger activation of eNOS may occur independently of cholesterol-rich microdomains. Interestingly, shear stress-induced eNOS phosphorylation was enhanced in cholesterol-enriched cells, but this did not correlate with enhanced eNOS activity. Hence, we propose that highly ordered and/or cholesterol-enhanced domains may contribute to promote shear stress-induced eNOS phosphorylation, but this may not be sufficient to drive eNOS activity under these conditions. We propose that cholesterol-enhanced domains have profound effects on HDL-induced eNOS activity, shear-stress-induced eNOS phosphorylation, but contribute less to VEGF-inducible eNOS activity. The molecular identify of these domains is still unclear, but based on experiments presented here, these domains are unlikely to be caveoale and could be Cav1-independent lipid raft domains [61]. Cholesterol also played a role in clustering of overexpressed Cav1 and here, cholesterol enrichment and Cav1 overexpression may lead to the formation of new caveolae. The fact that that shear stress can induce disassembly of caveolae, which does not occur with VEGF or HDL, further point at the requirement for different domains to ensure eNOS activation in response to different physiological stimuli.

In summary, this study, using for the first time cholesterol or 7KC enrichment rather than cholesterol depletion, supports a model for endothelial cells where VEGF, HDL and shear stress induced signalling cascades to trigger eNOS activation and phosphorylation display differential sensitivity towards cholesterol, possibly due to their location in distinct membrane domains.

## Materials and Methods

### Cell culture and sterol treatments

Bovine aortic endothelial cells (BAEC; passage 3–9, Cell Systems) were cultured in EBM (Endothelial basal medium) supplemented with EGM SingleQuot (Lonza), or in DMEM supplemented with 10% FBS, 50 U penicillin, 50 μg/ml streptomycin and 2 mM L-glutamine at 37°C with 5% CO_2_ until 70–80% confluent. Prior to experiments, BAEC were incubated in serum reduced DMEM containing 0.5–1.0% FBS, 5 U penicillin, 5 μg/ml streptomycin and 0.2 mM L-glutamine (serum-reduced DMEM) for a minimum of 2 h and maximum 16 h at 37°C with 5% CO_2_ and 95% air.

BAEC were enriched with 40 μM cholesterol, or 20 μM 7-ketocholesterol or a mixture of 20 μM cholesterol: 20 μM 7-ketocholesterol (1:1) for 30 min at 37°C with 5% CO_2_. Each sterol was complexed to 38 μM methyl-ß-cyclodextrin (mßCD) by adding 4 x 10 μL of 15 mg/mL cholesterol or 7-ketocholesterol (in water) to 400 μL of 5% mßCD (in water) at 80°C over the course of 1 h. To determine sterol enrichment of BAEC, the cells were lysed in 1 ml of 0.2 M NaOH. Samples were prepared for HPLC quantification as described previously [62].

### Stimulation with VEGF, HDL and shear stress

After sterol enrichment, the cells were stimulated by incubation with 25 ng/ml VEGF_165_ (VEGF; R&D Systems) for 5 min in serum-deprived media for most experiments or in Hepes/Krebs buffer for eNOS activity measurements. The cells were incubated in the presence of VEGF for 5 min at 37°C, 5% CO_2_.

HDL was isolated from human plasma by density gradient centrifugation. Cells were stimulated with 50 μg/ml HDL for 20 min before use for further experiments.

For shear stress experiments, sterilised microscopy slides were coated with a thin layer of 1% BD Matrigel in PBS and allowed to air dry for 2 h. The slides were rinsed once with PBS before use. BAEC were cultured on the slides in complete media until approximately 80–90% confluent, followed by serum-deprivation for a minimum of 2 h in serum-reduced DMEM. Shear stress was carried out in a Streamer model (Flexcell Inc.) parallel flow chamber at 10 dynes/cm^2^ for 5 min using serum-reduced DMEM as the flow medium.

### eNOS activity

BAECs were grown to confluency and incubated with or without 1 mM of the eNOS inhibitor L-NAME (L-nitro-arginine methyl ester) for 30 min in the presence or absence of sterol. All reagents were diluted in Hepes/Krebs buffer (composition in mmol/L: NaCl 99.01, KCl 4.69, CaCl_2_ 1.87, MgSO_4_ 1.20, K_2_HPO_4_ 1.03, NaHCO_3_ 25.0, Na-HEPES 20.0, and glucose 11.1; pH 7.4). To measure stimulated eNOS activity, cells were stimulated as described above. After stimulation or immediately after L-NAME incubation (basal activity) cells eNOS activity was assessed by quantification of the conversion of [^3^H]-L-arginine to [^3^H]-L-citrulline by liquid scintillation counting. Data is reported as the extent of conversion of [^3^H]-L-arginine to [^3^H]-L-citrulline that is sensitive to pre-treatment of the endothelial cell for 30 min with L-NAME (1 mM) and is expressed as dpm per 10^6^ endothelial cells.

### Immunoblotting

Primary antibodies were mouse anti- caveolin-1 (#610406), rabbit anti-caveolin-1 (#610060) and mouse anti-NOSIII (#610296), all from BD Transduction Laboratories. Rabbit anti-phospho-eNOS^Ser1179^ (#07–428) was from Upstate. Secondary antibodies used for immuno-blotting were anti-mouse horseradish peroxidase (anti-ms-HRP) and anti-rabbit horseradish peroxidase (anti-rb-HRP) (all from Jackson ImmunoResearch). Equal protein amounts (3–5 μg) per sample were loaded and separated using the NuPAGE (Invitrogen) systems. Separated proteins were transferred to nitrocellulose membranes (Amersham) using the NuPAGE transfer system or the iBlot Gel transfer system (Invitrogen). Proteins were detected with anti-ms-HRP or anti-rb-HRP secondary antibodies. Immunoblotted proteins were developed with ECL chemiluminescent western blotting system (Amersham) and imaged on a LAS-4000 mini system (Fujifilm). Protein chemiluminescent signals were quantified using the NIH image software (ImageJ) and expressed as arbitrary units (AU).

### Confocal and Laurdan imaging

Cells were seeded on gelatine-coated coverslips, enriched with sterols as described above and fixed with 4% paraformaldehyde (PFA) at room temperature. Excess PFA was quenched with 50 mM NH_4_Cl. Cells were permeabilised and blocked in blocking buffer (0.1% saponin, 0.5% BSA) for 1 h. To determine the subcellular distribution of Cav1 with eNOS cells were immunostained with Cav1-Alexa fluor 647 and eNOS-Alexa fluor 488.

For quantification of membrane order, cells were stained with 5 μM Laurdan for 20–30 min before fixation. After fixation and blocking, the cells were immunostained with Cav1-Cy3 and mounted using a mixture of Mowiol 4–88 and glycerol with 5% DABCO. Image acquisition was performed on a Leica Microsystems TCS SP5 microscope with a 1.4 NA 100x oil objective. Cav1-Alexa fluor 647 was excited with a DPSS laser at He/Ne laser at 633nm laser and eNOS-Alexa fluor 488 was excited with an Argon laser. Laurdan was excited at 800 nm with a MaiTai HP laser, Spectra-Physics (Leica Microsystems SP). Intensities were recorded with internal photon multiplier tubes. GP values were quantified as described by Owen et al. [53]. For quantification of Cav1-positive membrane areas, intensity images of Cav1-Cy3 were used to mask the GP images. The entire perimeter of the cells was outlined by selecting 5 pixels (equalling 1.2 μm) from the cell periphery and the GP quantified to determine total membrane order.

For comparing plasma membrane intensity against cytoplasm intensity in different sterol treatments, Cav1-Alexa fluor 647 and eNOS-Alexa fluor 488 intensity images were used to calculate co-localisation based on Pearson’s correlation. The plasma membrane and cytoplasm mean intensity was measured with Fiji ImageJ. The plasma membrane was represented by a 1–1.2 μm band traced around the perimeter of the cell and the area adjacent to the band represented intracellular regions. The mean average of the plasma membrane and cytoplasm were measured and compared.

### Transmission electron microscopy (TEM)

BAEC were treated as above, rinsed, fixed with 2% glutaraldehyde in 0.1 M phosphate buffer (PB) at 4°C for a minimum of 2 h and processed for TEM imaging. Samples were stained with with 1% OsO_4_ and dehydrated with acetone/PB, followed by embedding in Spurr resin (Sigma). The samples were cut into thin sections with an Ultramicrotome (Leica) and stained with uranyl acetate and lead citrate before being placed on copper grids. Cells were visualised on a Jeol JEM 1010 transmission electron microscope fitted with a Gatan Bioscan digital camera to record images or a Jeol JEM 1400 fitted with a Gatan Bioscan digital camera. Images were taken at magnifications of 25,000X or 40,000X. At these magnifications, caveolae structures were clearly visible on the plasma membrane, however it was not possible to image whole cells and thus it was necessary to image regions along the plasma membrane. Individual images of regions of the cell were connected using an Image J plugin called MosaicJ (NIH) to form a complete cell. To quantify caveolae/μm of cell perimeter, the whole cell perimeter in μm was mapped out using ImageJ, and caveolae counted manually along the cell perimeter.

### Photoactivated localisation microscopy (PALM) and analysis

BAEC were transiently transfected with Cav1-mEOS2 using Lipofectamine LTX and fixed with 4% PFA at 37°C. PALM images were acquired on a total internal reflection microscope (ELYRA PS-1, Carl Zeiss MicroImaging) with a 100x NA 1.46 oil-immersion objective. For mEos2, green-to-red photoconversion was achieved with 8 μW of 405-nm laser radiation, and red-converted mEos2 was imaged with 12 mW of 561-nm light. 15,000 images (exposure time 30 ms) were acquired per sample with a cooled, electron-multiplying charge-coupled device camera (iXon DU-897D; Andor).

Raw fluorescence intensity images were analysed with the software Zen 2010D (Carl Zeiss Microscopy). For each sample drift during acquisition was corrected relative to the position of surface-immobilized 100-nm colloidal gold beads (BBInternational).

After Gaussian and Laplace filtering, events were judged to have originated from single molecules when *I* − *M* > 6*S*, where *I* is event intensity, *M* is mean image intensity and *S* the standard deviation of image intensity. The centre of each point-spread function was then calculated by fitting intensity profiles to a two-dimensional Gaussian distribution. After drift correction, the *x*-*y* particle coordinates of each molecule were saved in a table. Two-dimensional molecular coordinates were cropped into non-overlapping regions of 3 μm × 3 μm in size, and events with localization precision worse than 30 nm discarded. Individual fluorophores can undergo several ‘blinking-cycles’, hence multiple blinks were accounted for by selection of an appropriate off-gap, as published previously [63, 64].

### Direct stochastic optical reconstruction microscopy (dSTORM) and analysis

Immuno-staining for STORM imaging were performed as described above for confocal imaging. In addition paraformaldehyde was quenched with 50 mM NH_4_Cl for 10min immediately after fixation. Cells were stained with Cav1 and Alexa Fluor 647 secondary antibodies and washed with PBS five times for 15 minutes per wash on a rocker.

dSTORM images were acquired with the same instrument as described for PALM. In order to excite and switch-off the fluorescent dye, Cav1-Alexa flour 647 was excited and switched off with the 642-nm excitation laser at maximum power (150 mW). Once the excited molecules were switched off the excitation laser was reduced to 45 mW and 20,000 frames (exposure time 30 ms and gain 50 V) were acquired in order to obtain super-resolution images. During acquisition, the 405-nm laser (between 0.05–2.5mW) was used to further activate more molecules when blinking rate was low.

The composition of the STORM buffer used for Alexa Fluor 647 includes: 25 mM HEPES, 25 mM glucose, 5% glycerol, pH 8.0, 10 mM 2-Mercaptoethylamine (SIGMA #30070), 50 μg/ml glucose oxidase (SIGMA #G2133) and 25 μg/ml of horseradish peroxidase (SIGMA #P8375). As with PALM, drift correction was performed with fiducials and the Zeiss software Zen 2010D software. dSTORM data analysis was performed as described for PALM data analysis.

### Statistics

The statistical significance multiple comparisons were made with one-way analysis of variance (ANOVA) and Tukey’s multiple comparisons test (Prism, Graphpad). The Pearson’s correlation was calculated with the JACoP plugin in ImageJ (NIH).
